# Subtoxic levels of hydrogen peroxide induce brain-derived neurotrophic factor expression to protect PC12 cells

**DOI:** 10.1186/1756-0500-7-840

**Published:** 2014-11-25

**Authors:** Yurina Ogura, Kazunori Sato, Ken-Ichi Kawashima, Nanako Kobayashi, Sei Imura, Kotaro Fujino, Hideo Kawaguchi, Taku Nedachi

**Affiliations:** Department of Life Sciences, Graduate School of Life Sciences, Toyo University, 1-1-1 Izumino, Itakura-machi, Oura-gun, Gunma, 374-0193 Japan; Faculty of Life Sciences, Toyo University, 1-1-1 Izumino, Itakura-machi, Oura-gun, Gunma, 374-0193 Japan

**Keywords:** Oxidative stress, BDNF, PC12 cells, Cell death

## Abstract

**Background:**

Oxidative stress is one of the mechanisms underlying pathogenesis in neurodegenerative diseases such as Alzheimer’s disease. Generally, oxidative stress represents cell toxicity; however, we recently found that oxidative stress promotes the expression of growth factor progranulin (*PGRN*) in HT22 murine hippocampus cells, thereby protecting the HT22 cells. In this study, we attempted to clarify whether a similar system exists in the other neuronal cell model, rat pheochromocytoma (PC12) cells.

**Results:**

After confirming that high concentrations of hydrogen peroxide (H_2_O_2_; 100–250 μM) initiate PC12 cell death, we analyzed growth factor expressional changes after H_2_O_2_ treatment. We found, intriguingly, that gene expression of brain-derived neurotrophic factor (*BDNF*), but not *PGRN* was significantly induced by H_2_O_2_. Although little expression of the high affinity BDNF receptor tropomyosin-related kinase TrkB was observed in PC12 cells, expression of low affinity neurotrophin receptor, p75NTR, was clearly observed. This BDNF signaling appeared to contribute to PC12 cell protection, since PC12 cell death was significantly attenuated by BDNF treatment.

**Conclusions:**

Based on our results, we conclude that the induction of BDNF by subtoxic levels of H_2_O_2_ and its signaling may have roles in PC12 cell protection.

## Background

Oxidative stress is generated by increases in reactive oxygen species (ROS) produced in the mitochondria and often has toxic effects on cell functions[1]. In the central nervous system (CNS), numerous reports suggest that oxidative stress is involved in the progression of some neurodegenerative diseases, such as Alzheimer’s disease (AD), Parkinson’s disease, and amyotrophic lateral sclerosis (ALS)[2–4]. Hence, understanding how oxidative stress enhances neuronal cell toxicity and exploring methods to control oxidative stress in the CNS are extremely important.

Neurotrophins such as nerve growth factors (NGF), brain-derived neurotrophic factor (BDNF), and neurotrophin-3 (NT-3), contribute to neuronal cell protection against oxidative stress[5]. Activation of mitogen-activated protein kinase (MAPK) and phosphatidylinositol 3-kinase (PI3K) cascades by these neurotrophic growth factors appears to have a central role in cell protection[6]. These two signaling cascades are generally activated via tropomyosin-related kinase (Trk) receptors that have tyrosine kinase activity (TrkA for NGF, TrkB for BDNF, and TrkC for NT-3, respectively). These neurotrophins have a different type of receptor, p75 neurotrophin receptor (p75NTR), whose role is less clear than that of Trk receptors. p75NTR belongs to the tumor necrosis factor receptor (TNFR) superfamily, and possesses similar ligand-dependent signaling pathways to TNFRs[7, 8]. Two major pathways activated by p75NTR are well documented: nuclear factor κB (NF-κB) pathway and Jun kinase (JNK) pathway[6]. Evidence suggests that the activation of the NF-kB pathway by p75NTR engagement promotes cell survival, whereas activation of JNK pathway promotes apoptosis[6]. These two distinct signaling pathways have completely opposite bio-effects, making the interpretation of physiological function of p75NTR signaling difficult.

The expression of neurotrophins from neuronal cells was regulated by multiple distinct stimulations. Recently, we found that subtoxic levels of oxidative stress significantly promoted the expression of a neurotrophic factor, progranulin (*PGRN*), in HT22 murine hippocampal cells[9]. Intriguingly, the expressed PGRN appeared to serve as autocrine/paracrine factor that had neuroprotective roles[9]. Based on this previous work, we hypothesized that when a neuronal cell experiences high levels of stress, the cell activates this autocrine/paracrine mechanism to protect itself and other cells. Whether similar autocrine/paracrine mechanisms, which are activated by subtoxic levels of oxidative stress, exist in other cell types is not well understood. The differentiated rat pheochromocytoma (PC12) cells have neuron-like characteristics[10], and thereby are often used for studying neuroprotection[11, 12]. Using the PC12 model, we determined whether subtoxic levels of oxidative stress activated observable neurotropic factor-mediated autocrine/paracrine cell protective mechanisms.

## Methods

### Materials

The western blot detection kit (ECL plus or ECL prime detection reagents) was from GE Healthcare Inc. (Rockford, IL, USA). Dulbecco’s Modified Eagle Medium (DMEM), penicillin/streptomycin and Trypsin-EDTA were purchased from Nakaraitesque (Kyoto, Japan). Cell culture equipment was from BD Biosciences (San Jose, CA, USA). Calf Serum (CS) and Fetal Bovine Serum (FBS) were obtained from BioWest (Nuaille, France). Immobilon-P was from Millipore Corp. (Bedford, MA, USA). Unless otherwise noted, all chemicals were of the purest grade available from Nakaraitesque, Sigma Chemicals (St. Louis, MO, USA) or Wako Pure Chemical Industries, Ltd. (Osaka, Japan). Because only an established cell line (PC12 cell) was used in this study, the ethics approval was not required.

### Cell culture

An established rat adrenal pheochromocytoma cell line, PC12 cell, was obtained from Dr. Shin-Ichiro Takahashi (The University of Tokyo, Tokyo, Japan). The PC12 cells were maintained in DMEM containing 10% FBS, 30 μg/ml penicillin, 100 μg/ml streptomycin at 37°C under a 5% CO_2_ atmosphere. The medium was exchanged every 72 h. For all experiments, cells were grown on 6-well plates (Corning Inc., Corning, NY, USA) at a density of 5 × 10^4^ cells/well in 3 ml of growth medium, or on 96-well plates (Corning Inc.) at a density of 5 × 10^3^ cells/well in 0.2 ml of growth medium. Three days after plating, cells typically reached 50-70% confluence (Day 0). Differentiation was then induced by switching to DMEM supplemented with 100 ng/ml NGF, 30 μg/ml penicillin, and 100 μg/ml streptomycin.

### Measurement of cell death

PC12 cells were seeded on 96-well plates and differentiated as described previously[13]. The percentage of cell death was evaluated using the lactose dehydrogenase (LDH) plus kit (Roche Diagnostics K.K., Basel, Switzerland) according to the manufacturer’s protocol.

### Western blotting

The expression and phosphorylation of each protein were analyzed by western blot analysis as described previously[13]. Briefly, the cells were seeded on 6-well plates at a density of 1 × 10^5^ cells/well, and 24 h later, the cells were treated with different concentrations of hydrogen peroxide (H_2_O_2_) for 30 min. The cell lysates were prepared using lysis buffer (2% sodium dodecyl sulfate (SDS), 1% 2-mercaptoethanol, 10% glycerol, 0.0033% Bromophenol Blue and 50 mM Tris–Cl [pH 6.8]). These cell lysates were resolved to 12% SDS-polyacrylamide gel electrophoresis (1:30, bis:acrylamide). Proteins were transferred to a polyvinylidene difluoride (PVDF) membrane (Immobilon-P; Millipore Corp, Bedford, MA, USA), and the membranes were blocked for 30 min at 3% bovine serum albumin (BSA) in tris buffered saline (TBS) containing 0.1% Tween-20. Detection of each protein was achieved with 1 h incubation with a 1:1000 dilution of primary antibody (anti-phospho Akt (S473), anti-Akt, anti-phospho Erk1/2, anti-Erk1/2, anti-phospho JNK, anti-JNK, anti-phospho p38, anti-p38, anti-IκB antibodies (Cell Signaling Technology, Danvers, MA, USA)). Specific total proteins were visualized after subsequent incubation with a 1:5000 dilution of anti-mouse or rabbit IgG conjugated to horseradish peroxidase and an ECL plus detection procedure (GE Healthcare Inc., Buckinghamshire, UK). At least three independent experiments were performed for each condition.

### PCR

RNA isolation from differentiated PC12 cells was performed using a Blood/Cultured Cell Total RNA mini kit (Favorgen Biotech Corp., Taiwan). The extracted total RNA was subjected to reverse transcriptase reaction using a PrimeScript real-time PCR (RT-PCR) kit (TAKARA, Osaka, Japan). PCR was performed using KAPATaq EXtra HotStart ReadyMix with dye (KAPA Biosystems Inc., Woburn, MA, USA) and the following PCR primers: rat TrkA, 5′-ATG CTC GTC AGG ACT TCC ATC G-3′ and 5′-TAG CCA CAG CCA GAA GCT GC-3′; rat TrkB, 5′-AAG TCC TCT ATG AAG ACT GGA CC-3′ and 5′-TGC CAA ACT TGG AAT GTC TCG CCA-3′; rat TrkC, 5′-CAG CCC AGA GCC TTT GCT AAG-3′ and 5′-GGC AAA GGA GAG CCA GAG CCA TT-3′; rat p75NTR, 5′-CGG AAT TCG GAG ACA TGT TCC ACA GGC-3′ and 5′-CCT TGG GAT CCA TCG ACC-3′.

### Real-time PCR

PC12 cells were differentiated as described previously, then treated with different concentrations of H_2_O_2_ (0–250 μM) for 12 h. Total RNA was isolated from cells using a High Pure RNA Isolation Kit (Roche Diagnostics, Mannheim, Germany) according to the manufacturer’s protocol. cDNAs were synthesized from total RNA using ReverTra Ace qPCR RT Master Mix (TOYOBO, Osaka, Japan). Fluorescence RT-PCR analysis was performed using a StepOne instrument (Life Technologies Corporation; Grand Island, NY, USA) and an SYBR Green detection kit according to the manufacture’s protocol (Life Technologies or KAPA Biosystems Inc.; Woburn, MA, USA). PCR primers for measuring each gene included the following: rat BDNF, 5′-TCA AGC TGG AAG CCT GAA TGA A-3′ and 5′-GCC AGT CAG GTA ACC ACT AAC AC-3′; rat PGRN, 5′-CAC TGT CCT GAT GGC TAC TCT TG-3′ and 5′-CTA CCA GGA CAC TGG ACA GCA C-3′; and rat GAPDH, 5′-GGC ACA GTC AAG GCT GAG AAT G-3′ and 5′-ATG GTG GTG AAG ACG CCA GTA-3′.

### Statistical analysis

Comparisons among treatment groups were tested using one-way ANOVA with Tukey’s post-tests. Differences for which p <0.05 were considered statistically significant.

## Results and discussion

### Subtoxic levels of oxidative stress induces BDNF expression

**Figure 1:**
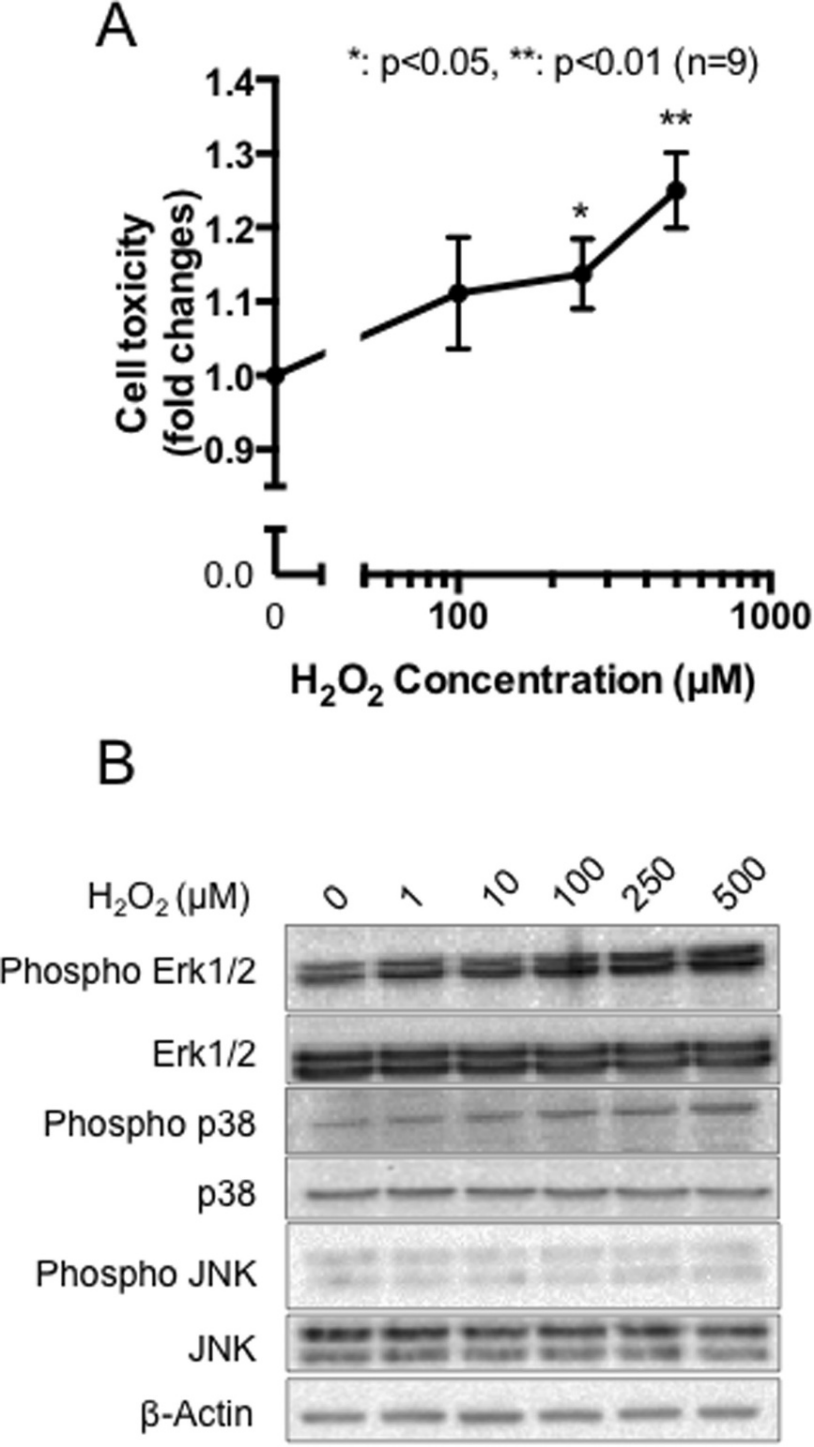
**BDNF expression is induced by subtoxic levels of oxidative stress in PC12 cells. (A)** Differentiated PC12 cells were stimulated with the indicated concentrations of H_2_O_2_ for 15 h. Cell toxicity was measured by LDH assay. Data shown represent mean ± SEM, tested using a one-way ANOVA with Tukey’s post-test (**p <0.01, n =9). **(B)** Differentiated PC12 cells were stimulated with the indicated concentration of H_2_O_2_ for 30 min. Total and phosphorylated proteins were evaluated by western blotting analysis. Three independent experiments were performed and representative data are shown.

Initially, we confirmed whether the treatment of PC12 cells with H_2_O_2_ induced cell death. As shown in Figure 1A, PC12 cell death gradually increased upon H_2_O_2_ treatment, and significant induction of cell death (p <0.05) was observed when more than 250 μM of H_2_O_2_ was applied to cells (Figure 1A). Moreover, as shown in Figure 1B, H_2_O_2_ treatment induced phosphorylation of stress-activated MAP kinases, Erk1/2 and p38, in a concentration-dependent manner, although the other MAP kinase, JNK, was not affected. From these data, we concluded that more than 250 μM of H_2_O_2_ indeed promoted oxidative stress to PC12 cells.

**Figure 2:**
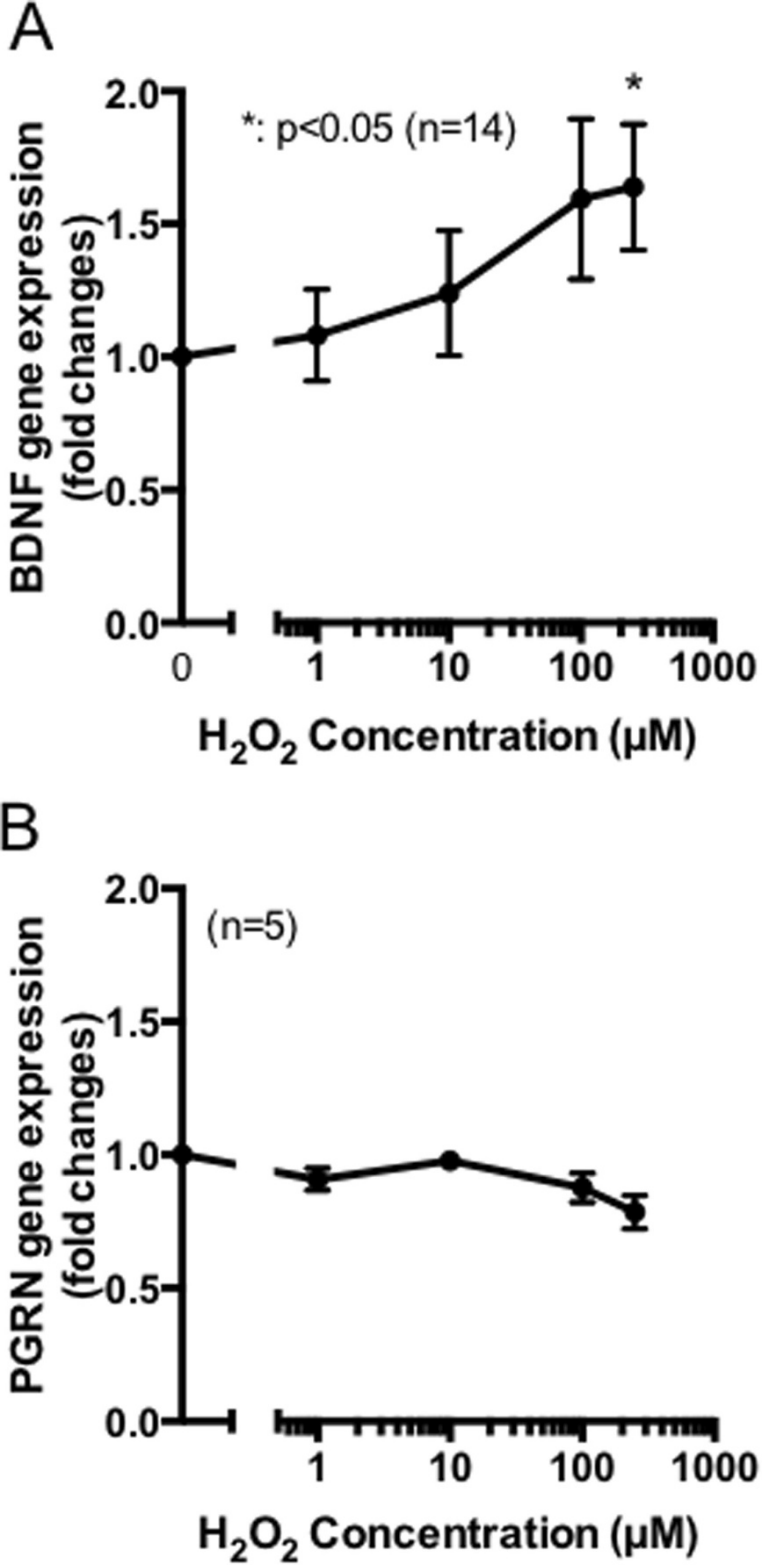
**Subtoxic levels of oxidative stress promote BDNF induction. (A, B)** Differentiated PC12 cells were stimulated with indicated concentrations of H_2_O_2_ for 15 h. Total RNA was then extracted from the cells and the gene expression of BDNF **(A)** or PGRN **(B)** was evaluated by quantitative PCR analysis. Data shown represent mean ± SEM, tested using one-way ANOVA with Tukey’s post-test (*p <0.05, n = 5–14).

As described in the background, we recently reported that subtoxic levels of oxidative stress significantly induced expression of a growth factor PGRN in HT22 murine hippocampal cells, thereby contributing to cell protection[9]. However, gene expression levels of *BDNF* and *IGF-1* were decreased by oxidative stress[9]. These results indicate that when the cells experienced subtoxic levels of oxidative stress, they activated specific intracellular machinery to promote specific growth factors. To investigate whether the expression of growth factors are similarly controlled in PC12 cells, we measured the gene expression of *PGRN*, *BDNF*, *NT3*, and *IGF1* after H_2_O_2_ treatment. Of these growth factors, only *BDNF* gene expression was induced in an H_2_O_2_ concentration-dependent manner (Figure 2A). Consistent with this result, Wang and colleagues report that BDNF secretion from differentiated PC12 cells was induced by hypoxic stimuli that were abolished by N-acetyl-l-cysteine, which is a scavenger of ROS[14]. Although the gene expression of *PGRN* was clearly observed in PC12 cells, its expression levels were not altered by H_2_O_2_ administration (Figure 2B). In addition, little gene expression of *NT3* and *IGF1* was observed (data not shown). Together, these results demonstrate that subtoxic levels of oxidative stress specifically promote *BDNF* expression in PC12 cells. Moreover, compared to similar experiments using HT22 cells that we previously reported[9], the oxidative stress-dependent regulation of growth factors appeared to be varied among neuronal cell types.

### BDNF signals through p75NTR in PC12 cells

**Figure 3:**
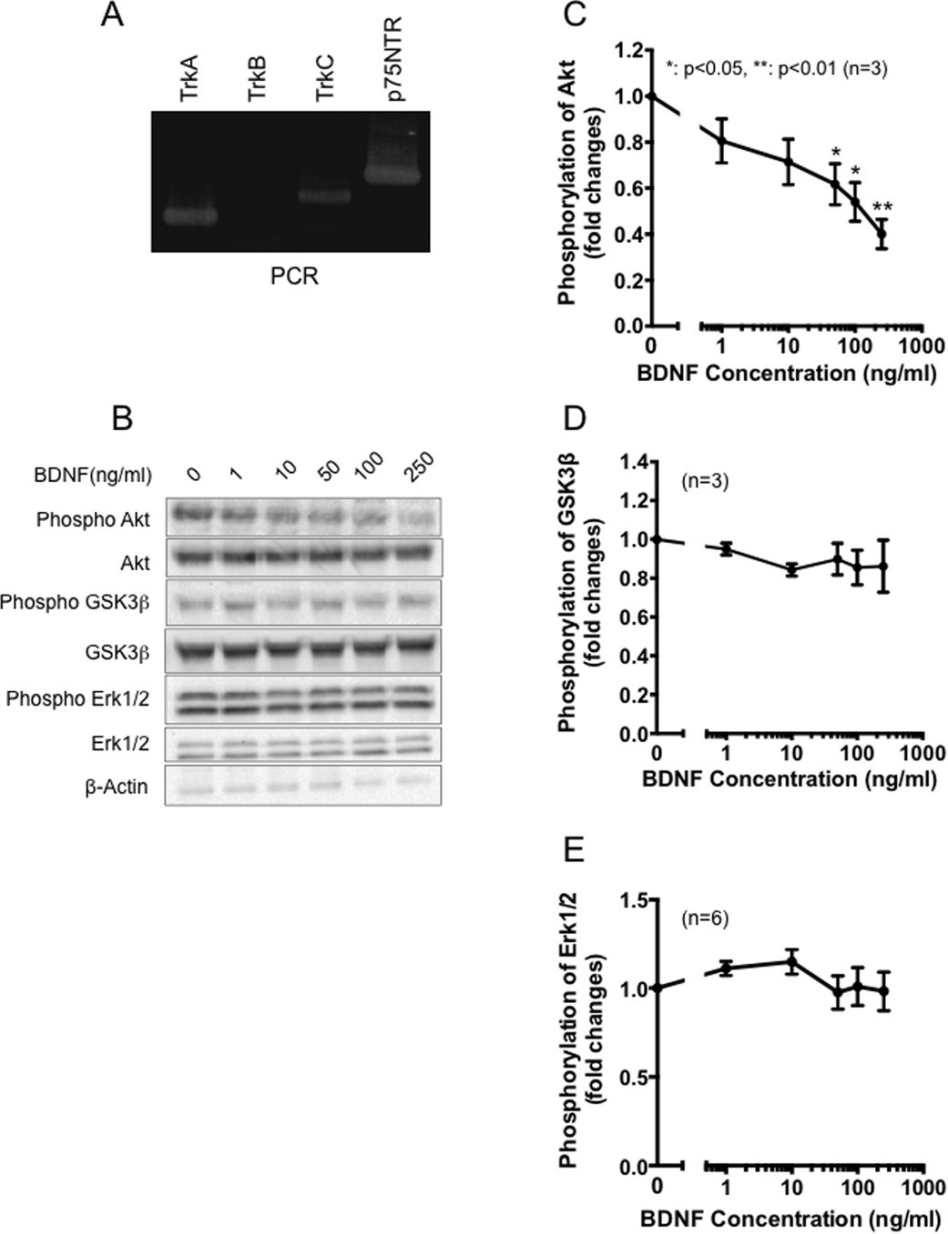
**BDNF signaling in PC12 cells. (A)** Total RNA was extracted from differentiated PC12 cells. The gene expression of each neurotrophin receptors was evaluated by RT-PCR analysis. **(B)** Differentiated PC12 cells were treated with the indicated amounts of BDNF for 30 min. Total and phosphorylated proteins were evaluated by western blotting analysis. Three independent experiments were performed and representative data are shown. **(C, D, E)** Densitometric analysis of **(B)**. Data shown represent mean ± SEM, tested using one-way ANOVA with Tukey’s post-test (*p <0.05, **p <0.01, n = 3).

Based on our hypothesis that BDNF induced by H_2_O_2_ may function in an autocrine/paracrine manner, we explored how PC12 cells responded against BDNF. Before evaluating the effects of BDNF on cellular functions, we analyzed whether BDNF receptor was expressed in PC12 cells, since it has been reported that high affinity BDNF receptor, TrkB, is not expressed in PC12 cells[15]. As shown in Figure 3A, we also confirmed that detectable levels of TrkB was not observed. On the contrary, TrkA, TrkC, and low affinity neurotrophin receptor p75NTR were expressed in PC12 cells (Figure 3A). BDNF-p75NTR signaling has been well studied and is especially characterized by prominent activation of NFκB signaling[16]. To test if BDNF treatment affects Trk signaling, we also analyzed Erk1/2 and Akt phosphorylation that are activated by the neurotrophin-Trk dependent signaling pathway. The amount of IκB, which inhibits NFκB nuclear translocation, was not changed by BDNF treatment (Figure 3B). The amounts and phosphorylation of NFκB were also not affected by BDNF (data not shown). In terms of Trk-dependent signaling, changes in Erk1/2 and GSK3β phosphorylation were not observed, but Akt phosphorylation was significantly decreased by BDNF treatment (Figure 3B–E). It was reported that pro-NGF induces expression of phosphatase and tensin homolog deleted on chromosome 10 (PTEN), a negative regulator for PI3K signaling, and thereby abolishes Akt activation in brain neurons[17]. However, our present results revealed that BDNF has an ability to dephosphorylate Akt acutely, within 30 min. Overall, our present data suggest that BDNF inactivated Akt perhaps via p75NTR, although little TrkB was expressed in PC12 cells.

### BDNF treatments protect PC12 cells

**Figure 4:**
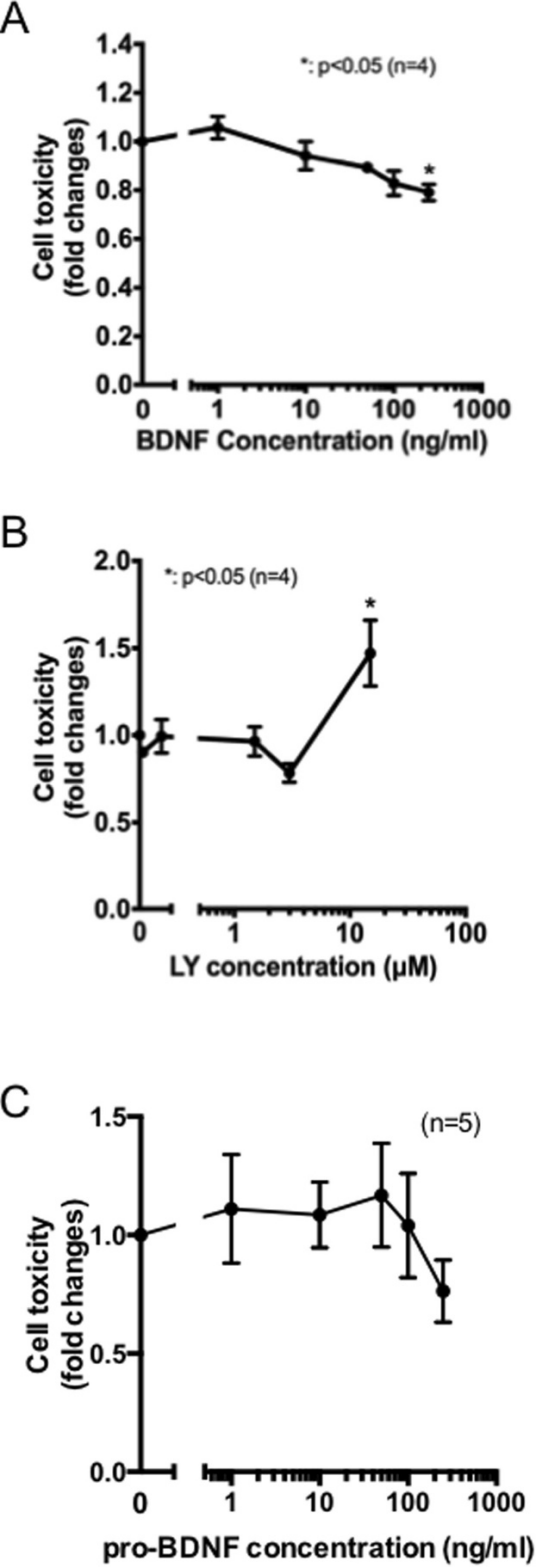
**BDNF protects PC12 cells from death. (A)** Differentiated PC12 cells were treated with the indicated amounts of BDNF for 15 h, and cell toxicity was measured by LDH assay. Data shown represent mean ± SEM, tested using a one-way ANOVA with Tukey’s post-tests (*p <0.05, n = 4). **(B)** Differentiated PC12 cells were treated with the indicated amounts of the PI3K inhibitor LY294002 for 15 h, and cell toxicity was measured by LDH assay. Data shown represent mean ± SEM, tested using one-way ANOVA with Tukey’s post-test (*p <0.05, n = 4). **(C)** Differentiated PC12 cells were treated with the indicated amounts of pro-BDNF for 15 h, and cell toxicity was measured by LDH assay. Data shown represent mean ± SEM.

BDNF treatment protects against various insults[18–20]; however, if these protections occur in cells that lack TrkB receptors, such as PC12 cells, is not well studied. To determine if BDNF protects PC12 cells in the absence of TrkB receptors, PC12 cells were treated with BDNF for 24 h and cell viability was evaluated by measuring released LDH (described in Method*s*). As shown in Figure 4A, BDNF treatment slightly but significantly ameliorated PC12 cell survival rate. Currently, we have not identified which signaling pathways mediate cell protective effects dependent on BDNF. Several studies suggest that p75NTR could promote cell survival by enhancing NGF signaling pathways[21–23]; however, we could not confirm that major NGF signaling pathways, the Erk1/2 and PI3K/Akt cascades, were enhanced by BDNF treatment. Rather, Akt phosphorylation was significantly decreased by the BDNF treatment (Figure 3B,3C). Pharmacological inhibition of the PI3K/Akt pathway by LY294002 enhanced PC12 cell death (Figure 4B), indicating that BDNF-dependent Akt inactivation might contribute to induction of cell death. It should be noted that the oxidative stress-dependent induction of *BDNF* gene might also result in enhancement of pro-BDNF production. Although whether pro-BDNF has a physiological function has been controversial[24, 25], recent reports suggest that pro-BDNF preferentially binds to p75NTR, and exerts pro-apoptotic effects[26]. However, intriguingly, exogenous pro-BDNF treatment also tended to reduce PC12 cell death (Figure 4C). Thus, although further experiments are required to measure the concentration of mature BDNF and pro-BDNF in the vicinity of cells, the induction of BDNF gene by H_2_O_2_ treatment appears to be beneficial for PC12 cells.

**Figure 5:**
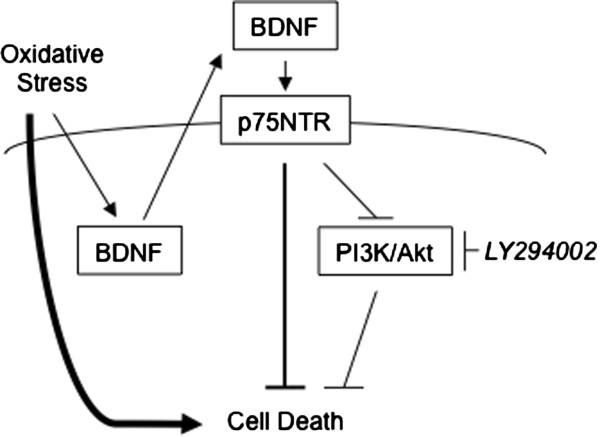
**Schematic depiction of the present study.** Oxidative stress generated by H_2_O_2_ treatment induces PC12 cell death; however, this subtoxic level of oxidative stress also induces BDNF. BDNF signals via p75NTR to protect PC12 cells, even though it inactivates the PI3K/Akt cascade that potentially induces cell survival.

Overall, our results suggest that subtoxic levels of oxidative stress promote *BDNF* gene expression, and possibly exert a cell protective mechanism (Figure 5). Consistent with our present observations, several recent studies suggest that ROS accumulation could exert beneficial effects on adaptation against stress and survival of cells[27, 28]. We found that although the BDNF-dependent signaling pathway possesses cell protective functions, it perhaps inactivates PI3K/Akt pathway that appeared to be a negative factor for cell survival. Our laboratory is now exploring the other signaling pathway that is activated by BDNF and is crucial for cell protection. An intriguing observation from the present study is that oxidative stress and BDNF potentially activate both cell death and cell survival promoting mechanisms. The balance between these two opposing systems may directly influence the determination of cellular fates.

## Conclusions

Subtoxic levels of oxidative stress induce *BDNF* gene expression that potentially exerts a cell protective mechanism.

## Abbreviations

BSA: Bovine serum albumin
DMEM: Dulbecco’s modified Eagle’s medium
LDH: Lactate dehydrogenase
NGF: Nerve growth factor
PCR: Polymerase chain reaction
PBS: Phosphate-buffered saline
TBS: Tris-buffered saline.
